# 2-(3,4-Dichloro­phen­yl)-4-phenyl­benzo[*h*]quinoline

**DOI:** 10.1107/S1600536811040049

**Published:** 2011-10-05

**Authors:** Nan Wu, Zhou Xu

**Affiliations:** a Department of Aviation Oil and Materials, Xuzhou Airforce College, Xuzhou Jiangsu 221110, People’s Republic of China; b Department of Chemistry, Xuzhou Medical College, Xuzhou Jiangsu 221004, People’s Republic of China

## Abstract

In the title compound, C_25_H_15_Cl_2_N, the benzo[*h*]quinoline system exhibits an approximately planar conformation with an r.m.s. deviation of 0.0202Å and a maximum deviation of 0.039 (1) Å. The aryl group at position 2 is nearly coplanar with the parent ring [dihedral angle = 6.68 (7)°] while the parent ring and the phenyl subsitituent at position 4 form a dihedral angle of 67.11 (4)°. Inter­molecular C—H⋯π inter­actions stabilize the crystal packing.

## Related literature

For the uses of metal complexes of benzo[*h*]quinoline as electronic materials and organic electronic devices, see: Cho *et al.* (2010 ▶). For the medicinal uses of benzo[*h*]quinoline and its complexes, see: Pantoom *et al.* (2011 ▶); Liu *et al.* (2011 ▶). For the preparation of the title compound, see: Zhang *et al.* (2010 ▶).
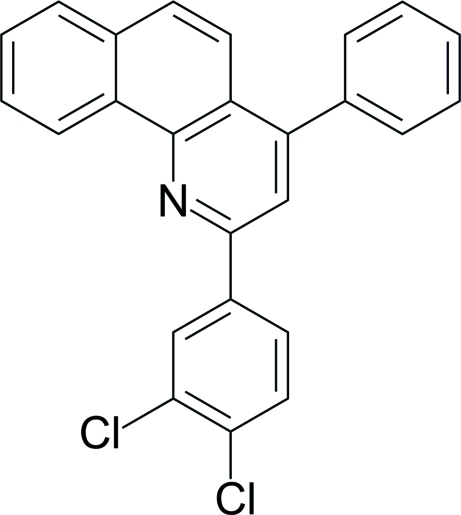


## Experimental

### 

#### Crystal data

C_25_H_15_Cl_2_N
*M* *_r_* = 400.28
Monoclinic, 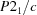

*a* = 10.6066 (14) Å
*b* = 9.5667 (12) Å
*c* = 18.824 (2) Å
β = 94.264 (7)°
*V* = 1904.8 (4) Å^3^
*Z* = 4
Mo *K*α radiation
μ = 0.35 mm^−1^
*T* = 113 K
0.20 × 0.18 × 0.12 mm

#### Data collection

Rigaku Saturn724 CCD diffractometer
Absorption correction: multi-scan (*CrystalClear*; Rigaku/MSC, 2002) ▶ *T*_min_ = 0.933, *T*_max_ = 0.959
23687 measured reflections
4523 independent reflections
3630 reflections with *I* > 2σ(*I*)
*R*_int_ = 0.045

#### Refinement

*R*[*F* ^2^ > 2σ(*F* ^2^)] = 0.039
*wR*(*F* ^2^) = 0.110
*S* = 1.07
4523 reflections
253 parameters
H-atom parameters constrained
Δρ_max_ = 0.37 e Å^−3^
Δρ_min_ = −0.36 e Å^−3^

### 

Data collection: *CrystalClear* (Rigaku/MSC, 2002) ▶; cell refinement: *CrystalClear* ▶; data reduction: *CrystalClear* ▶; program(s) used to solve structure: *SHELXS97* (Sheldrick, 2008 ▶); program(s) used to refine structure: *SHELXL97* (Sheldrick, 2008 ▶); molecular graphics: *SHELXTL* (Sheldrick, 2008 ▶); software used to prepare material for publication: *SHELXTL*.

## Supplementary Material

Crystal structure: contains datablock(s) I, global. DOI: 10.1107/S1600536811040049/hg5101sup1.cif

Structure factors: contains datablock(s) I. DOI: 10.1107/S1600536811040049/hg5101Isup2.hkl

Supplementary material file. DOI: 10.1107/S1600536811040049/hg5101Isup3.cml

Additional supplementary materials:  crystallographic information; 3D view; checkCIF report

## Figures and Tables

**Table 1:** Hydrogen-bond geometry (Å, °) *Cg*1, *Cg*2 and *Cg*3 are the centroids of the C20–C25, C14–C19 and N1/C1/C10–C13 rings, respectively.

*D*—H⋯*A*	*D*—H	H⋯*A*	*D*⋯*A*	*D*—H⋯*A*
C6—H6⋯*Cg*1^i^	0.95	2.98	3.8577 (19)	154
C22—H22⋯*Cg*2^ii^	0.95	2.94	3.8204 (19)	156
C25—H25⋯*Cg*3^iii^	0.95	2.63	3.4738 (17)	148

Symmetry codes: (i) 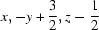; (ii) 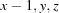; (iii) 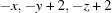.

**Fig. 1.**
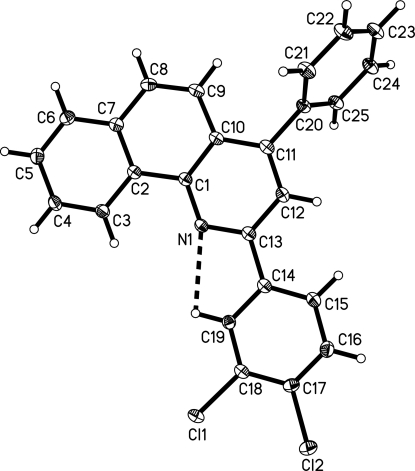
The structure of (I), showing 30% probability displacement ellipsoids and the atom-numbering scheme. Cg1 is the centroid of the ring of C20/C21/C22/C23/C24/C25. Cg2 is the centroid of the ring of C14/C15/C16/C17/C18/C19. Cg3 is the centroid of the ring of N1/C1/C10/C11/C12/C13.

**Fig. 2.**
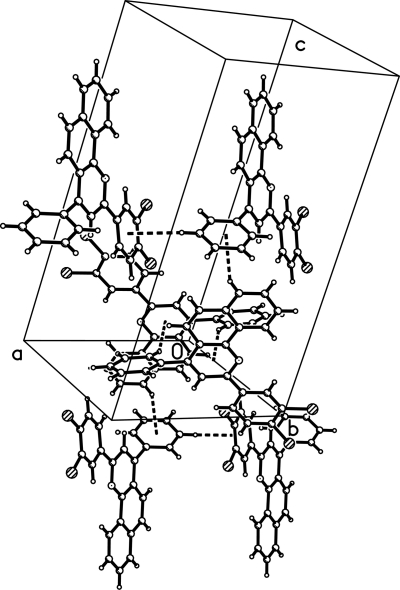
The packing diagram of (I).

## supplementary crystallographic information

### Comment

The benzo[*h*]quinoline derivatives and their complexes can be used as electronic material and organic electronic device (Cho *et al.* ,2010), potent family-18 chitinase inhibitors (Pantoom *et al.*, 2011), topoisomerase IIα poisons (Liu *et al.*, 2011). Besides, They can also treat Alzheimer's disease. These properties arouse our interset in the relationship between their structures and activities. During the synthesis of benzo[*h*]quinoline derivatives, the title compound, (I) was isolated and its structure was determined by X-ray diffraction. Herein we shall report its crystal structure. The molecular structure of (I) is shown in Fig. 1. In the molecular structure, the benzo[*h*]quinoline exhibits a planar conformation with RMS of 0.0202Å and the largest deviation is 0.039 (1) Å. The 3,4-dichlorophenyl is almost coplanar with benzo[*h*]quinoline, since the dihedral angle between them is only 6.68 (7)°. The parent ring and the phenyl subsitituent at position 4 form a dihedral angle of 67.11 (4)°. In addition, there is a non-classical intramolecular hydrogen bond (C19—H19···N1). The crystal packing is stabilized by the intermolecular C—H···π interactions (Fig. 2, Table 1).

### Experimental

The title compound was synthesized according to the reported procedure (Zhang *et al.*, 2010). Under an air atmosphere, a 10 ml of sealable reaction tube equipped with a magnetic stir bar was charged with an 3,4-dichlorobenzaldehyde (1.00 mmol), naphthalen-1-amine (1.00 mmol), and the mixture was heated and stirred in an oil bath at 333 K for 1 h. Then FeCl_3_ (16.2 mg, 0.10 mmol), ethynylbenzene (1.10 mmol) were added. The reaction mixture was then stirred in an oil bath at 393 K until the substrates were consumed completely (about 12 h), and then it was cooled to room temperature and the solvent was evaporated, the residue was purified by flash chromatography(hexane/AcOEt = 15:1) to afford the desired product.The single-crystal suitable for X-ray diffraction was obtained through the evaporation of ethanol solution.

### Refinement

All H atoms were placed in calculated positions, with C—H = 0.95 Å, and included in the final cycles of refinement using a riding model, with Uĩso~(H) = 1.2U~eq~(parent atom).

### Crystal data

**Table:**

C_25_H_15_Cl_2_N	*F*(000) = 824
*M**_r_* = 400.28	*D*_x_ = 1.396 Mg m^−^^3^
Monoclinic, *P*2_1_/*c*	Mo *K*α radiation, λ = 0.71073 Å
Hall symbol: -P 2ybc	Cell parameters from 6694 reflections
*a* = 10.6066 (14) Å	θ = 1.9–27.9°
*b* = 9.5667 (12) Å	µ = 0.35 mm^−^^1^
*c* = 18.824 (2) Å	*T* = 113 K
β = 94.264 (7)°	Prism, colorless
*V* = 1904.8 (4) Å^3^	0.20 × 0.18 × 0.12 mm
*Z* = 4	

### Data collection

**Table:**

Rigaku Saturn724 CCD diffractometer	4523 independent reflections
Radiation source: rotating anode	3630 reflections with *I* > 2σ(*I*)
multilayer	*R*_int_ = 0.045
Detector resolution: 14.22 pixels mm^-1^	θ_max_ = 27.8°, θ_min_ = 1.9°
ω and φ scans	*h* = −13→13
Absorption correction: multi-scan (*CrystalClear*; Rigaku/MSC, 2002)	*k* = −12→12
*T*_min_ = 0.933, *T*_max_ = 0.959	*l* = −24→24
23687 measured reflections	

### Refinement

**Table:**

Refinement on *F*^2^	Primary atom site location: structure-invariant direct methods
Least-squares matrix: full	Secondary atom site location: difference Fourier map
*R*[*F*^2^ > 2σ(*F*^2^)] = 0.039	Hydrogen site location: inferred from neighbouring sites
*wR*(*F*^2^) = 0.110	H-atom parameters constrained
*S* = 1.07	*w* = 1/[σ^2^(*F*_o_^2^) + (0.0602*P*)^2^] where *P* = (*F*_o_^2^ + 2*F*_c_^2^)/3
4523 reflections	(Δ/σ)_max_ = 0.002
253 parameters	Δρ_max_ = 0.37 e Å^−^^3^
0 restraints	Δρ_min_ = −0.36 e Å^−^^3^

### Special details

**Table:**

Geometry. All e.s.d.'s (except the e.s.d. in the dihedral angle between two l.s. planes) are estimated using the full covariance matrix. The cell e.s.d.'s are taken into account individually in the estimation of e.s.d.'s in distances, angles and torsion angles; correlations between e.s.d.'s in cell parameters are only used when they are defined by crystal symmetry. An approximate (isotropic) treatment of cell e.s.d.'s is used for estimating e.s.d.'s involving l.s. planes.
Refinement. Refinement of *F*^2^ against ALL reflections. The weighted *R*-factor *wR* and goodness of fit *S* are based on *F*^2^, conventional *R*-factors *R* are based on *F*, with *F* set to zero for negative *F*^2^. The threshold expression of *F*^2^ > σ(*F*^2^) is used only for calculating *R*-factors(gt) *etc*. and is not relevant to the choice of reflections for refinement. *R*-factors based on *F*^2^ are statistically about twice as large as those based on *F*, and *R*- factors based on ALL data will be even larger.

### Fractional atomic coordinates and isotropic or equivalent isotropic displacement parameters (Å^2^)

**Table:**

	*x*	*y*	*z*	*U*_iso_*/*U*_eq_	
Cl1	0.52078 (4)	0.42736 (4)	1.09062 (2)	0.03323 (13)	
Cl2	0.56082 (4)	0.57108 (5)	1.24136 (2)	0.03876 (14)	
N1	0.09940 (11)	0.64203 (12)	0.98050 (6)	0.0220 (3)	
C1	−0.00408 (13)	0.66067 (15)	0.93439 (8)	0.0217 (3)	
C2	−0.00284 (14)	0.59559 (15)	0.86472 (8)	0.0226 (3)	
C3	0.10040 (15)	0.51461 (17)	0.84545 (8)	0.0270 (4)	
H3	0.1729	0.5043	0.8778	0.032*	
C4	0.09621 (16)	0.45062 (16)	0.78003 (9)	0.0310 (4)	
H4	0.1656	0.3950	0.7678	0.037*	
C5	−0.00939 (16)	0.46621 (17)	0.73071 (9)	0.0318 (4)	
H5	−0.0110	0.4210	0.6857	0.038*	
C6	−0.11019 (16)	0.54695 (16)	0.74773 (9)	0.0292 (4)	
H6	−0.1807	0.5586	0.7141	0.035*	
C7	−0.10939 (14)	0.61255 (16)	0.81487 (8)	0.0239 (3)	
C8	−0.21411 (14)	0.69559 (16)	0.83422 (8)	0.0260 (3)	
H8	−0.2849	0.7073	0.8008	0.031*	
C9	−0.21506 (14)	0.75764 (15)	0.89867 (8)	0.0264 (3)	
H9	−0.2853	0.8136	0.9092	0.032*	
C10	−0.11070 (13)	0.73999 (15)	0.95172 (8)	0.0231 (3)	
C11	−0.10774 (14)	0.79943 (15)	1.02076 (8)	0.0236 (3)	
C12	−0.00148 (14)	0.78015 (16)	1.06638 (8)	0.0249 (3)	
H12	0.0027	0.8206	1.1126	0.030*	
C13	0.10125 (14)	0.70060 (15)	1.04485 (8)	0.0229 (3)	
C14	0.21694 (14)	0.67568 (15)	1.09325 (8)	0.0231 (3)	
C15	0.23628 (14)	0.73938 (16)	1.15973 (8)	0.0291 (4)	
H15	0.1761	0.8048	1.1745	0.035*	
C16	0.34251 (14)	0.70838 (17)	1.20477 (8)	0.0311 (4)	
H16	0.3546	0.7529	1.2499	0.037*	
C17	0.43053 (14)	0.61299 (17)	1.18414 (8)	0.0276 (4)	
C18	0.41328 (14)	0.55001 (15)	1.11722 (8)	0.0250 (3)	
C19	0.30761 (14)	0.58159 (15)	1.07252 (8)	0.0238 (3)	
H19	0.2967	0.5385	1.0270	0.029*	
C20	−0.21559 (14)	0.88347 (16)	1.04496 (8)	0.0245 (3)	
C21	−0.33198 (14)	0.82296 (18)	1.05638 (9)	0.0328 (4)	
H21	−0.3450	0.7260	1.0476	0.039*	
C22	−0.42876 (16)	0.90333 (19)	1.08044 (9)	0.0361 (4)	
H22	−0.5078	0.8614	1.0881	0.043*	
C23	−0.41049 (16)	1.04477 (17)	1.09328 (9)	0.0335 (4)	
H23	−0.4769	1.0996	1.1100	0.040*	
C24	−0.29624 (16)	1.10611 (18)	1.08182 (9)	0.0330 (4)	
H24	−0.2842	1.2034	1.0899	0.040*	
C25	−0.19865 (15)	1.02549 (16)	1.05835 (8)	0.0274 (4)	
H25	−0.1195	1.0678	1.0514	0.033*	

### Atomic displacement parameters (Å^2^)

**Table:**

	*U*^11^	*U*^22^	*U*^33^	*U*^12^	*U*^13^	*U*^23^
Cl1	0.0260 (2)	0.0342 (2)	0.0393 (3)	0.01057 (17)	0.00126 (18)	−0.00259 (18)
Cl2	0.0285 (2)	0.0490 (3)	0.0374 (3)	0.00308 (19)	−0.00704 (18)	−0.0028 (2)
N1	0.0228 (7)	0.0176 (6)	0.0260 (7)	0.0012 (5)	0.0046 (5)	0.0016 (5)
C1	0.0220 (8)	0.0166 (7)	0.0269 (8)	−0.0008 (6)	0.0039 (6)	0.0018 (6)
C2	0.0223 (8)	0.0188 (7)	0.0270 (8)	−0.0010 (6)	0.0047 (6)	0.0022 (6)
C3	0.0245 (8)	0.0254 (8)	0.0312 (9)	0.0021 (7)	0.0023 (7)	−0.0014 (7)
C4	0.0302 (9)	0.0294 (9)	0.0339 (9)	0.0042 (7)	0.0059 (7)	−0.0066 (7)
C5	0.0343 (9)	0.0322 (9)	0.0292 (9)	−0.0019 (8)	0.0046 (7)	−0.0052 (7)
C6	0.0278 (9)	0.0296 (9)	0.0298 (9)	−0.0028 (7)	−0.0002 (7)	0.0016 (7)
C7	0.0232 (8)	0.0202 (8)	0.0287 (8)	−0.0026 (6)	0.0038 (6)	0.0033 (6)
C8	0.0214 (8)	0.0260 (8)	0.0306 (8)	−0.0008 (6)	0.0006 (6)	0.0066 (7)
C9	0.0210 (7)	0.0234 (8)	0.0354 (9)	0.0028 (6)	0.0055 (7)	0.0040 (7)
C10	0.0205 (7)	0.0194 (7)	0.0298 (8)	0.0005 (6)	0.0055 (6)	0.0032 (6)
C11	0.0234 (8)	0.0184 (7)	0.0297 (8)	0.0003 (6)	0.0078 (6)	0.0038 (6)
C12	0.0281 (8)	0.0215 (8)	0.0259 (8)	0.0024 (6)	0.0072 (6)	0.0007 (6)
C13	0.0238 (8)	0.0173 (7)	0.0279 (8)	−0.0009 (6)	0.0051 (6)	0.0027 (6)
C14	0.0239 (8)	0.0188 (7)	0.0272 (8)	−0.0008 (6)	0.0054 (6)	0.0022 (6)
C15	0.0288 (9)	0.0267 (8)	0.0323 (9)	0.0042 (7)	0.0050 (7)	−0.0027 (7)
C16	0.0329 (9)	0.0313 (9)	0.0291 (9)	−0.0019 (7)	0.0024 (7)	−0.0055 (7)
C17	0.0235 (8)	0.0284 (9)	0.0302 (9)	−0.0026 (7)	−0.0022 (7)	0.0029 (7)
C18	0.0228 (8)	0.0214 (8)	0.0314 (9)	0.0013 (6)	0.0060 (7)	0.0008 (7)
C19	0.0256 (8)	0.0217 (8)	0.0242 (8)	0.0004 (6)	0.0030 (6)	0.0001 (6)
C20	0.0246 (8)	0.0245 (8)	0.0249 (8)	0.0049 (6)	0.0052 (6)	0.0037 (6)
C21	0.0291 (9)	0.0256 (9)	0.0447 (10)	−0.0001 (7)	0.0105 (8)	0.0016 (7)
C22	0.0276 (9)	0.0366 (10)	0.0458 (11)	0.0022 (8)	0.0142 (8)	0.0046 (8)
C23	0.0307 (9)	0.0345 (10)	0.0368 (10)	0.0113 (8)	0.0129 (7)	0.0052 (8)
C24	0.0345 (9)	0.0256 (9)	0.0399 (10)	0.0085 (7)	0.0096 (8)	0.0018 (7)
C25	0.0252 (8)	0.0253 (8)	0.0324 (9)	0.0021 (7)	0.0073 (7)	0.0036 (7)

### Geometric parameters (Å, °)

**Table:**

Cl1—C18	1.7356 (15)	C12—C13	1.413 (2)
Cl2—C17	1.7347 (16)	C12—H12	0.9500
N1—C13	1.3334 (18)	C13—C14	1.492 (2)
N1—C1	1.3595 (19)	C14—C15	1.393 (2)
C1—C10	1.420 (2)	C14—C19	1.394 (2)
C1—C2	1.453 (2)	C15—C16	1.391 (2)
C2—C3	1.411 (2)	C15—H15	0.9500
C2—C7	1.423 (2)	C16—C17	1.382 (2)
C3—C4	1.373 (2)	C16—H16	0.9500
C3—H3	0.9500	C17—C18	1.396 (2)
C4—C5	1.409 (2)	C18—C19	1.384 (2)
C4—H4	0.9500	C19—H19	0.9500
C5—C6	1.376 (2)	C20—C25	1.391 (2)
C5—H5	0.9500	C20—C21	1.395 (2)
C6—C7	1.411 (2)	C21—C22	1.385 (2)
C6—H6	0.9500	C21—H21	0.9500
C7—C8	1.435 (2)	C22—C23	1.386 (2)
C8—C9	1.351 (2)	C22—H22	0.9500
C8—H8	0.9500	C23—C24	1.378 (2)
C9—C10	1.444 (2)	C23—H23	0.9500
C9—H9	0.9500	C24—C25	1.389 (2)
C10—C11	1.417 (2)	C24—H24	0.9500
C11—C12	1.378 (2)	C25—H25	0.9500
C11—C20	1.497 (2)		
			
C13—N1—C1	118.79 (12)	N1—C13—C14	116.32 (13)
N1—C1—C10	122.84 (14)	C12—C13—C14	121.84 (14)
N1—C1—C2	117.34 (13)	C15—C14—C19	118.38 (14)
C10—C1—C2	119.82 (13)	C15—C14—C13	122.55 (14)
C3—C2—C7	119.10 (14)	C19—C14—C13	119.04 (14)
C3—C2—C1	121.79 (14)	C16—C15—C14	120.85 (14)
C7—C2—C1	119.10 (13)	C16—C15—H15	119.6
C4—C3—C2	120.10 (14)	C14—C15—H15	119.6
C4—C3—H3	119.9	C17—C16—C15	120.21 (15)
C2—C3—H3	119.9	C17—C16—H16	119.9
C3—C4—C5	121.00 (15)	C15—C16—H16	119.9
C3—C4—H4	119.5	C16—C17—C18	119.52 (14)
C5—C4—H4	119.5	C16—C17—Cl2	120.12 (12)
C6—C5—C4	119.97 (15)	C18—C17—Cl2	120.36 (12)
C6—C5—H5	120.0	C19—C18—C17	120.01 (14)
C4—C5—H5	120.0	C19—C18—Cl1	119.47 (12)
C5—C6—C7	120.35 (15)	C17—C18—Cl1	120.48 (12)
C5—C6—H6	119.8	C18—C19—C14	121.01 (14)
C7—C6—H6	119.8	C18—C19—H19	119.5
C6—C7—C2	119.45 (14)	C14—C19—H19	119.5
C6—C7—C8	121.38 (14)	C25—C20—C21	118.78 (14)
C2—C7—C8	119.18 (14)	C25—C20—C11	119.24 (14)
C9—C8—C7	121.92 (14)	C21—C20—C11	121.95 (14)
C9—C8—H8	119.0	C22—C21—C20	120.39 (16)
C7—C8—H8	119.0	C22—C21—H21	119.8
C8—C9—C10	120.86 (14)	C20—C21—H21	119.8
C8—C9—H9	119.6	C21—C22—C23	120.11 (16)
C10—C9—H9	119.6	C21—C22—H22	119.9
C11—C10—C1	117.49 (13)	C23—C22—H22	119.9
C11—C10—C9	123.43 (13)	C24—C23—C22	120.12 (15)
C1—C10—C9	119.08 (14)	C24—C23—H23	119.9
C12—C11—C10	118.65 (13)	C22—C23—H23	119.9
C12—C11—C20	119.37 (14)	C23—C24—C25	119.88 (16)
C10—C11—C20	121.98 (13)	C23—C24—H24	120.1
C11—C12—C13	120.39 (14)	C25—C24—H24	120.1
C11—C12—H12	119.8	C24—C25—C20	120.71 (15)
C13—C12—H12	119.8	C24—C25—H25	119.6
N1—C13—C12	121.83 (13)	C20—C25—H25	119.6
			
C13—N1—C1—C10	0.0 (2)	C1—N1—C13—C14	179.61 (12)
C13—N1—C1—C2	179.91 (13)	C11—C12—C13—N1	0.0 (2)
N1—C1—C2—C3	0.8 (2)	C11—C12—C13—C14	−179.01 (13)
C10—C1—C2—C3	−179.29 (14)	N1—C13—C14—C15	174.74 (13)
N1—C1—C2—C7	179.98 (13)	C12—C13—C14—C15	−6.2 (2)
C10—C1—C2—C7	−0.1 (2)	N1—C13—C14—C19	−7.5 (2)
C7—C2—C3—C4	−1.5 (2)	C12—C13—C14—C19	171.63 (14)
C1—C2—C3—C4	177.67 (14)	C19—C14—C15—C16	−0.9 (2)
C2—C3—C4—C5	1.1 (2)	C13—C14—C15—C16	176.90 (14)
C3—C4—C5—C6	0.2 (2)	C14—C15—C16—C17	−0.3 (2)
C4—C5—C6—C7	−1.0 (2)	C15—C16—C17—C18	1.2 (2)
C5—C6—C7—C2	0.6 (2)	C15—C16—C17—Cl2	−178.31 (13)
C5—C6—C7—C8	−179.10 (15)	C16—C17—C18—C19	−1.0 (2)
C3—C2—C7—C6	0.7 (2)	Cl2—C17—C18—C19	178.52 (11)
C1—C2—C7—C6	−178.51 (13)	C16—C17—C18—Cl1	−178.77 (12)
C3—C2—C7—C8	−179.61 (14)	Cl2—C17—C18—Cl1	0.77 (19)
C1—C2—C7—C8	1.2 (2)	C17—C18—C19—C14	−0.2 (2)
C6—C7—C8—C9	179.24 (15)	Cl1—C18—C19—C14	177.60 (12)
C2—C7—C8—C9	−0.4 (2)	C15—C14—C19—C18	1.1 (2)
C7—C8—C9—C10	−1.4 (2)	C13—C14—C19—C18	−176.76 (13)
N1—C1—C10—C11	−1.0 (2)	C12—C11—C20—C25	65.91 (19)
C2—C1—C10—C11	179.08 (13)	C10—C11—C20—C25	−113.34 (17)
N1—C1—C10—C9	178.24 (13)	C12—C11—C20—C21	−112.36 (17)
C2—C1—C10—C9	−1.7 (2)	C10—C11—C20—C21	68.4 (2)
C8—C9—C10—C11	−178.35 (14)	C25—C20—C21—C22	0.2 (2)
C8—C9—C10—C1	2.5 (2)	C11—C20—C21—C22	178.44 (15)
C1—C10—C11—C12	1.5 (2)	C20—C21—C22—C23	0.1 (3)
C9—C10—C11—C12	−177.71 (14)	C21—C22—C23—C24	0.3 (3)
C1—C10—C11—C20	−179.25 (13)	C22—C23—C24—C25	−0.9 (3)
C9—C10—C11—C20	1.5 (2)	C23—C24—C25—C20	1.2 (2)
C10—C11—C12—C13	−1.1 (2)	C21—C20—C25—C24	−0.8 (2)
C20—C11—C12—C13	179.67 (13)	C11—C20—C25—C24	−179.11 (14)
C1—N1—C13—C12	0.5 (2)		

### Hydrogen-bond geometry (Å, °)

**Table:**

Cg1, Cg2 and Cg3 are the centroids of the C20–C25, C14–C19 and N1/C1/C10–C13 rings, respectively.

**Table:**

*D*—H···*A*	*D*—H	H···*A*	*D*···*A*	*D*—H···*A*
C6—H6···Cg1^i^	0.95	2.98	3.8577 (19)	154.
C22—H22···Cg2^ii^	0.95	2.94	3.8204 (19)	156.
C25—H25···Cg3^iii^	0.95	2.63	3.4738 (17)	148.
C19—H19···N1	0.95	2.42	2.765 (2)	101.

Symmetry codes: (i) *x*, −*y*+3/2, *z*−1/2; (ii) *x*−1, *y*, *z*; (iii) −*x*, −*y*+2, −*z*+2.
